# Mapping DNA Topoisomerase Binding and Cleavage Genome Wide Using Next-Generation Sequencing Techniques

**DOI:** 10.3390/genes11010092

**Published:** 2020-01-13

**Authors:** Shannon J. McKie, Anthony Maxwell, Keir C. Neuman

**Affiliations:** 1 Laboratory of Single Molecule Biophysics, NHLBI, Bethesda, MD 20892, USA; Shannon.McKie@jic.ac.uk; 2 Department of Biological Chemistry, John Innes Centre, Norwich NR4 7UH, UK; tony.maxwell@jic.ac.uk

**Keywords:** DNA topoisomerase, next-generation sequencing, genome wide, topoisomerase cleavage, topoisomerase binding, antibiotics, anticancer drugs

## Abstract

Next-generation sequencing (NGS) platforms have been adapted to generate genome-wide maps and sequence context of binding and cleavage of DNA topoisomerases (topos). Continuous refinements of these techniques have resulted in the acquisition of data with unprecedented depth and resolution, which has shed new light on in vivo topo behavior. Topos regulate DNA topology through the formation of reversible single- or double-stranded DNA breaks. Topo activity is critical for DNA metabolism in general, and in particular to support transcription and replication. However, the binding and activity of topos over the genome in vivo was difficult to study until the advent of NGS. Over and above traditional chromatin immunoprecipitation (ChIP)-seq approaches that probe protein binding, the unique formation of covalent protein–DNA linkages associated with DNA cleavage by topos affords the ability to probe cleavage and, by extension, activity over the genome. NGS platforms have facilitated genome-wide studies mapping the behavior of topos in vivo, how the behavior varies among species and how inhibitors affect cleavage. Many NGS approaches achieve nucleotide resolution of topo binding and cleavage sites, imparting an extent of information not previously attainable. We review the development of NGS approaches to probe topo interactions over the genome in vivo and highlight general conclusions and quandaries that have arisen from this rapidly advancing field of topoisomerase research.

## 1. Introduction

DNA topoisomerases (topos), pervasive across all domains of life and indispensable for cellular survival, alter DNA topology through the formation of transient single- or double-stranded DNA breaks (DSBs); for comprehensive reviews, see [1,2]. Generally speaking, topos can relax both positive and negative supercoils, as well as unknot and decatenate interlinked DNA. Performing these reactions is vital to maintaining genomic integrity, particularly during DNA transcription, replication, and segregation. A common feature among all topos is the formation of a reversible covalent link between the protein and the DNA backbone, known as a cleavage complex. Whilst this is an integral step in the topo mechanism, it is also a highly vulnerable state for the duplex. Numerous topo poisoning agents exploit this vulnerability by binding at the cleaved DNA site and preventing re-ligation [3,4]. Other endogenous factors such as colliding replication/transcription forks or DNA lesions in close proximity, can also contribute to the cleavage complex becoming a stabilized DSB [5]. Using topo II inhibitors to treat cancer has implicated topos directly in the development of secondary cancers such as acute myeloid leukemia, which arises through genomic translocations caused by the attempted repair of inhibitor-stabilized topo-dependent DSBs [6,7].

**Figure 1:**
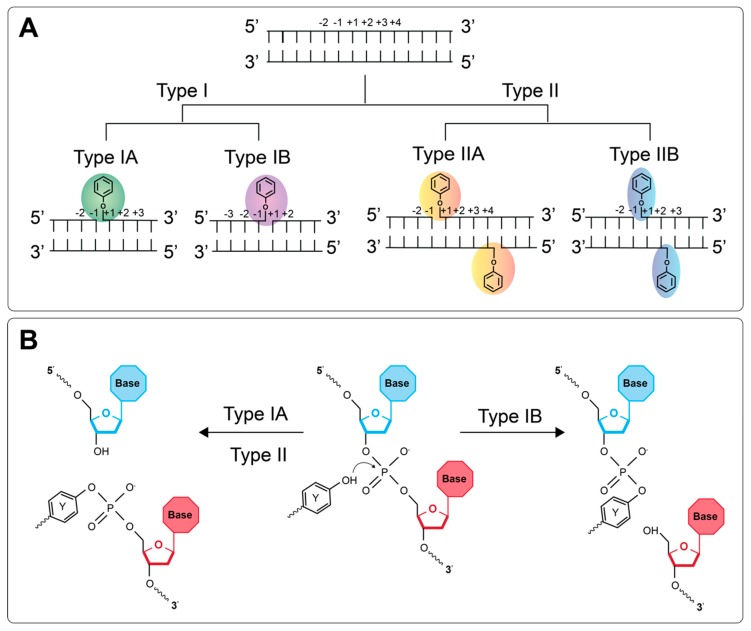
DNA cleavage by the DNA topoisomerases. (**A**) Representation of where the DNA is cleaved and the phophotyrosyl bond that is formed by each type of topo. (**B**) Chemistry of the covalent attachment between the active site tyrosine of the topo and the DNA backbone. For type IA and II topos, the 5′ phosphate group undergoes nucleophilic attack by the tyrosine OH group, whereas, for type IB, it is the 3′ phosphate group.

The cleavage complex formed when topos actively engage with the duplex differs amongst the different types (Figure 1). The type I topos, which form single-stranded breaks (SSBs), are subcategorized as type IA and type IB. Type IA topos form 5′ covalent bonds between the active site tyrosine and the 5′ phosphate group of the DNA backbone. The type IB topos differ in that the active site tyrosine attacks the 3′ phosphate group instead. The type II topos, which form DSBs, are also subcategorized as type IIA and type IIB. The type IIA topos form two 5′ covalent linkages between the active site tyrosines and the 5′ phosphate, one on each strand, offset by 4 base-pairs (bp), and therefore generating 4 bp 5′ overhangs. The type IIB topos behave similarly, except they are thought to produce 2 bp 5′ overhangs (see Figure 1 for details) [2].

As they are vital to cellular survival and constitute effective antibiotic and anti-cancer targets, understanding the DNA binding and cleavage profiles of topos in a genome-wide context is of great interest to the field. Whereas the fundamental biochemistry of the topo reactions has been established, how their activity is regulated across the genome and through time in vivo has only recently begun to be addressed in a systematic manner with the utilization of next-generation sequencing (NGS) technologies. In this review, we discuss the genome-wide mapping of topos that have been characterized using NGS approaches and the specific nuances of the protocols developed to do so (summarized in Table 1).

### 1.1. Next-Generation Sequencing

The first widely used sequencing technique was developed in 1977 by Frederick Sanger and colleagues, which employed the use of chain terminating dideoxynucleotides [20]. It was later automized by Applied Biosystems with the development of the AB370 in 1987, which accelerated sequencing time and increased accuracy by using capillary electrophoresis [21]. Whilst this was a revolutionary technique for the time, and facilitated the completion of the first human genome in 2004 [22], this ‘first generation’ sequencing method had its limitations. Sanger sequencing is relatively low throughput, time intensive, and expensive. Efforts to ameliorate sequencing resulted in the development of affordable, high throughput technologies. This led to a number of innovations in the DNA sequencing field and the birth of next-generation sequencing (NGS) platforms [23]. NGS platforms allow cell-free library preparation and are able to perform millions of parallel sequencing reactions, detecting the output directly without electrophoresis. This meant that genomes could be fully sequenced in an extraordinarily short timeframe. The main drawback, particularly for the first NGS platforms, was the relatively short read length. However, this was circumvented through the development of new algorithmic tools for aligning short reads.

In 2005 the first NGS platform was released by Roche, the 454 genome sequencer [24]. The detection method used, called pyrosequencing, relied on the activity of pyrophosphate, which would emit light when a base was incorporated, and allowed the sequencing of ~200,000 reads, ~110 bp long per run, generating 20 Mb of data. More recent advances made reads of 700 bp possible, generating 0.7 GB of data in less than 24 h with the 454 GS FLX Titanium system. In 2006, Sequencing by Oligo Ligation Detection (SOLiD) by Agencourt, was bought by Applied Biosystems [21]. This NGS platform uses octamer oligos, each with a unique fluorescent label, that interrogate the first two bases in the sequence adjacent to the sequencing primer. Once the label color is detected, it is released by cleavage between bases 5 and 6 of the octamer, and another probe will hybridize to the next two free bases and so on. The process is then repeated using a sequencing primer one nucleotide shorter than the first. In this manner, the first-round sequences bases in positions 4, 5, 9, 10, 14, 15, etc., whilst the second round sequences 3, 4, 8, 9, 13, 14, etc., until sequencing primer position 0 is reached [25]. Due to each base being interrogated twice, early SOLiD systems benefitted from high accuracy, with more recent versions able to sequence 85 bp fragments with 99.99% accuracy [21]. Also, in 2006, the Illumina platform was released, which facilitates detection through the use of fluorescently labelled reversible-terminator nucleotides. Each nucleotide is uniquely colored allowing its incorporation to be detected, before the subsequent removal of the 3′ terminator group along with the fluorescent probe. Numerous Illumina platforms have since been manufactured, employing this technique, such as MiSeq, HiSeq and NextSeq, with the main difference between these systems being the depth of parallel sequencing power and data output. The most recently released Illumina technology, Novaseq, is capable of performing up to 40 billion sequencing reactions in parallel, of up to 250 bp in length with an output maximum of 6000 Gb. This sequencing scale has opened the door to a wide range of innovative approaches that leverage these platforms to develop unique methodologies to probe field-specific questions. In this review, the research cited relies on using one of the three aforementioned platforms. In most instances, the choice of platform is somewhat arbitrary, largely dictated by the best available sequencing depth and length. A comprehensive overview of sequencing platforms is provided in [21].

### 1.2. Non-Specific DSB Mapping

Genome metabolism is highly coordinated, dynamic and staggeringly complex, with numerous proteins interacting with both the duplex and other proteins, to maintain DNA integrity while facilitating DNA processing. DNA within each cell is subject to an assault from both endogenous and exogenous factors, resulting in various forms of damage that require either repair or tolerance [26]. Arguably one of the more toxic forms of damage is the induction of DNA breaks, particularly DSBs, which can lead to carcinogenic translocations or cell death [27]. One early use of NGS was to explore the DSB landscape of the genome, to better understand when and where the majority of DSBs occur. One such study developed a generalized technique called BLESS (direct in situ Breaks Labelling, Enrichment on Streptavidin and next-generation Sequencing) [9]. By labelling DSBs in situ, the level of false positives caused by genome shearing during extraction was minimized, allowing the resolution of the “breakome” in both human and mouse cells. The cells were fixed and lysed to attain purified nuclei, which were then subject to biotinylated adapter ligation. The genomic DNA could then be fragmented and enriched for endogenously labelled DSBs using streptavidin. Exposing cells to aphidicolin, an inhibitor of DNA polymerase, it was found that replication stress is a major driver of DSB generation, particularly within genes and satellite regions. BLESS was also able to accurately identify telomere ends as well as Sce-1 endonuclease sites.

A subsequent study developed an alternative technique, designed to overcome noise and background complications associated with BLESS, as well as to gain information on DSB end structure [10]. Termed END-seq, this protocol involves embedding mouse thymocytes or lymphocytes within agarose before soaking the agarose in proteinase K solution, followed by an RNase solution. The agarose embedded cells are then end repaired and adapter ligated, using a specifically indexed and biotinylated adapter. The DNA is then extracted, sheared, and immunoprecipitated using streptavidin, before ligation of the second adapter. The DNA library was single-end sequenced using Illumina Hiseq2500 or Nextseq500. This technique proved to be highly sensitive, with nucleotide resolution at break sites, and led to a number of new insights in the field of T- and B-cell research. It also has potential as a tissue specific CRISPR-Cas9 off target cleavage detection protocol. A limitation, however, is evident in the end repair stage in which 3′ overhangs are resected and 5′ overhangs are filled-in to blunt the DNA for adapter ligation. This means that any information concerning the length of the overhang is lost.

### 1.3. Spo11 DSB Mapping

One of the first studies to implement the use of NGS in topoisomerase-specific genome-wide DSB mapping, explored the generation of meiosis specific DNA cleavage by the topoisomerase-related protein, Spo11, in *Saccharomyces cerevisiae* [12]. Spo11 acts as a homodimer and is highly homologous to the topoisomerase VIA subunit [28]. It is responsible for DSB generation during meiosis, which ultimately leads to chromosomal recombination, making Spo11 an important factor in driving evolution through the generation of genome diversity. Spo11 homodimers form covalent 5′ phosphotyrosine linkages on both strands of a DNA duplex, generating a DSB with a 2 bp overhang, a process highly reminiscent of the type II topoisomerases. The main difference being that Spo11 DSBs are irreversible and followed by endonucleolytic cleavage adjacent to the bound Spo11. This releases the Spo11 monomer covalently attached to an oligonucleotide (oligo) and generates 3′ overhangs, which facilitate strand invasion and recombination. The released Spo11-bound oligos are separated into two distinct subpopulations of equal quantity, longer (21–37 nt) and shorter (<12 nt). Pan and colleagues used these oligos to develop an NGS-based approach, termed Spo11-oligo-seq, designed to overcome limitations encountered by previous studies employing DNA microarray analysis, to attain a comprehensive genome-wide Spo11-mediated DSB map with nucleotide resolution at the cleavage sites.

**Figure 2:**
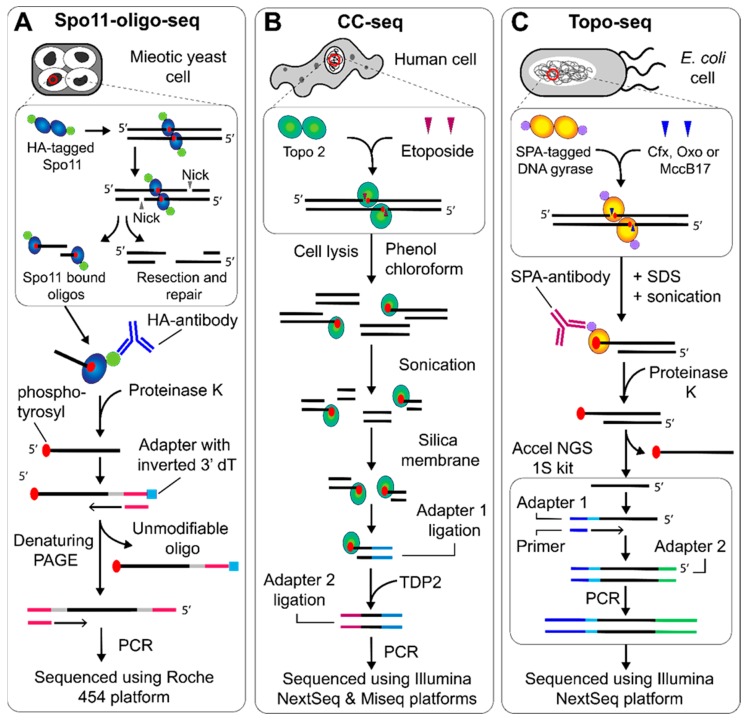
NGS protocols to map topo cleavage sites genome wide. (**A**) Spo11-oligo-seq began with DNA extraction from meiotic *S. cerevisiae* cells. HA-tagged Spo11-bound DNA oligos are enriched with the HA-antibody, before the protein is removed using proteinase K. The 3′ end is extended using TdT and an adapter is ligated that contains an inverted dT residue. The complementary strand is synthesized, followed by separation using denaturing PAGE, 3′ end tailing and adapter ligation on the newly synthesized strand, which can then be sequenced using the Roche 454 platform [12]. (**B**) CC-seq, used to map topo II cleavage, began with DNA extraction from etoposide treated human cells. The bulk protein is removed using phenol-chloroform extraction, whilst the protein bound to DNA remains in the aqueous phase. The DNA can then be sonicated and enriched using a silica membrane. The first ligation attached adapters to the sonicated end, which was then followed by TDP2-dependent removal of the topo II, before a second adapter ligation followed by sequencing using the Illumina NextSeq and Miseq platforms [15]. (**C**) Topo-seq, used to map gyrase cleavage, began with DNA extraction from *E. coli* cells treated with ciprofloxacin (cfx), oxolinic acid (oxo) or microcin B-17 (MccB17), followed by sonication. Gyrase cleavage complexes were then enriched using the SPA-antibody before proteinase K treatment and use in the Accel NGS 1S kit to generate fragments that can be sequenced using the Illumina Nextseq platform [18].

Nuclear extracts were obtained from synchronous meiotic yeast cells and the Spo11-bound oligos captured via immunoprecipitation and then released by proteolytic digestion (Figure 2A). The oligos were then 3′-GTP tailed using terminal deoxynucleotidyl transferase (TdT) and ligated to a double-stranded DNA adapter using T4 DNA ligase before the complementary strand was synthesized using Klenow polymerase. The DNA was purified using denaturing polyacrylamide gel electrophoresis (PAGE), before a second round of 3′-GTP end tailing, adapter ligation and strand synthesis. The original oligo cannot be further modified as the 3′ adapter has an inverted dT and the 5′ DNA end is blocked by the phosphotyrosine residue. Sequencing adapters were then added via PCR before the oligos were sequenced using the 454 platform (Roche). The fragments could then be aligned and bioinformatically processed, generating an in-depth representation of Spo11-DSB activity genome wide. This revealed a complex picture in which the DSB landscape in *S. cerevisiae* is influenced by numerous factors in a hierarchical manner, and that the concept of DSB ‘hotspots’ [29] is misleading. Spo11 demonstrates an opportunistic cutting activity where most sites can constitute a cleavage site but with variable probability that is modulated by numerous other factors.

### 1.4. DNA Topoisomerase II

In 2014, human topoisomerase II (topo II), both the α- and β-isoforms collectively became the first true topoisomerases explored at the genome-wide scale [14]. Human colon cancer cells (HCT116) were treated with etoposide (a topo II cleavage-complex stabilizing agent) and the genomic DNA extracted. Here, two different methods were implemented to capture both SSBs and DSBs, named SSB-seq and DSB-seq, respectively. In order to label SSBs, nick-translation was used, a technique first described in 1977, which enables labelling of DNA at a nick site [13]. In this case, digoxigenin-labelled dUTP was used so that the nicked DNA fragments, once sonicated, could be immunoprecipitated using anti-digoxigenin. For DSB-seq, the DNA was 3′-end tailed using TdT in the presence of biotinylated dNTPs so that after sonication, the DNA could be enriched using streptavidin coated beads. S1-nuclease was used to cleave the 3′-end tailing and then both the SSB- and DSB-seq DNA populations were adapter ligated and sequenced using Illumina.

This study highlighted the tendency of low-dose etoposide to produce SSBs, as opposed to DSBs, indicating that etoposide predominantly acts on a single monomer of topo II, suggesting in turn that only one cleavage site is poisoned, leaving the other to be freely re-ligated. This is in agreement with a previous study that found only 3% of etoposide-stabilized cleavages are DSBs [30]. It is worth noting, that whilst topo I (type IB) can form single-stranded nicks, these nicks do not have the free 3′-OH group, so are not substrates for nick-translation and won’t be labelled, making SSB-seq topo II specific. Whole-genome analysis revealed that topo II SSBs and DSBs were enriched at transcription start sites and more prevalent in highly expressed genes, further demonstrating the cellular necessity of topo II in regulating topology during transcription.

In 2016, came a comprehensive study looking specifically at murine liver topo IIβ activity, using ChIP-seq, ChIP-exo and Hi-C [31]. For an in-depth ChIP-seq protocol see [8], however, in short, DNA protein interactions were stabilized using a crosslinking agent and topo IIβ-bound DNA was enriched using anti-topo IIβ antibody. The crosslinking was reversed, and the DNA underwent library preparation in which the ends were repaired, A-tailed, and adapters were ligated before sequencing using the Illumina HiSeq2500 platform.

Topo IIβ and topo IIα, whilst being structurally and catalytically alike, are functionally distinct, performing different cellular functions. Topo IIβ knockouts are lethal owing to disruption of neuronal differentiation [32]. Conditional knockouts implicated topo IIβ activity in retinal development [33] and ovulation [34], and the use of poisons showed it played a role in spermatogenesis [35] and lymphocyte activation [36]. This has led to a picture of topo IIβ being an important factor in tissue-specific development and cellular differentiation, a concept elaborated upon by Uusküla-Reimand and colleagues with the help of ChIP-seq [31]. They found topo IIβ interacts with the transcriptional regulator CTCF and the cohesin complex and acts on DNA located at the borders of chromosomal domains as well as conserved transcription factor binding sites.

Topo IIα activity genome wide was also explored in depth in 2018 [37], using human leukemia K562 cells treated with etoposide, mitoxantrone, genistein or p-benzoquinone, all of which are known topo II inhibitors. DNA was extracted and sonicated before bead-based immunocapture using rabbit anti-topo IIα polyclonal antibody. The DNA was released from the topo IIα using calf alkaline phosphatase (CIP), an enzyme which is capable of phosphodiesterase activity at 3% of its canonical monophosphatase activity. CIP is therefore able to partially remove the phosphotyrosine residue, allowing for end repair and adapter ligation. The generated libraries were then sequenced using the illumina HiSeq2500 platform. In agreement with past studies, they found that the majority of topo IIα cleavage sites were SSBs rather than DSBs, the latter only making up 1.3–3.4% of cleavage events. Reads clustered in regions of compact genome, reminiscent of the cleavage profile of yeast Spo11, and with a preference for regions with high transcriptional activity. They also identified chromosomes that consistently contained more topo IIα cleavage sites than others. In particular chromosome 11 that includes the *KMT2A* gene, which houses a drug-induced leukemia translocation break point. The *KMT2A* gene, along with others, forms the *KMT2A* recombinome, and a proportion of these genes demonstrated significantly higher levels of topo IIα cleavage. They also concluded that topo IIα cleavage sites were enriched in genes necessary for DNA metabolism and transcriptional regulation.

As protocols are refined, an increasingly more precise DSB landscape for topo II is emerging. In 2019, DSB maps were generated with cleavage-site nucleotide resolution for human and yeast topo II [15]. The protocol, termed cleavage complex (CC)-seq (Figure 2B), begins with genomic DNA extraction from human or yeast cells treated with etoposide, followed by a phenol-chloroform extraction to remove bulk proteins, leaving protein-bound DNA in the aqueous fraction. The DNA is then sonicated and enriched using a silica membrane, which will only bind DNA that is protein bound. The library preparation then occurs in two phases, with the P7 adapter being ligated to the protein-free DNA end. The topo II is then cleaved using TDP2, an enzyme evolved to rescue stalled topo II cleavage complexes in vivo [38], before the P5 adapter is ligated and the library is sequenced. In this study the NGS platforms used were the Illumina MiSeq and NextSeq 500.

As this study was conducted on both human and yeast cell lines, species-specific conclusions about topo II activity could be made. For both human and yeast, topo II activity was increased in gene dense regions of the genome. However, only a correlation with transcription rate and gene length was observed in human cells. For yeast, topo II activity was concentrated in intergenic regions, which are numerous and evenly spaced. For human cells, topo II activity was found in the gene bodies just downstream of the transcription start site and was maximized for genes that are longer and more actively transcribed. This strengthens the hypothesis that topo II in human cells plays an integral role in topology maintenance during transcription. This gene-local activity pattern may not be necessary in yeast as the genome is both far smaller and less complex. There is also the potential alternative that in yeast cells topo I may be more active in relieving transcriptional stress on the duplex than topo II, whilst in human cells this role is more evenly shared. It is worth noting that for topo IIα specifically [37], activity in the absence of cleavage-complex stabilizing drugs was found to be located at the distal ends of genes, with the addition of drugs causing a proximal shift.

An analysis of etoposide-induced topo II cleavage complexes (topoIIcc) in mouse and human cell lines used the aforementioned END-seq protocol, which was adapted to explore both processed and unprocessed topoIIccs [11]. Mouse embryonic fibroblasts (MEFs) depleted of the cohesin subunit, SMC3, exhibited a dramatic decrease in topoIIcc, whereas Smc3^+/+^ control cells exhibited topoIIcc enrichment at cohesin sites. Not only was this decrease in topoIIcc proportional to coehsin levels in MEFs, similar results were obtained with a HCT-116 human cell line depleted of RAD21, another member of the cohesin complex. Moreover, depleting MEFs for the WAPL protein, responsible for cohesin removal from DNA, also increased the levels of etoposide-induced DNA damage. Interestingly, whilst topoII activity as well as binding (also measured here using ChIP-seq) was dependent on cohesin occupancy; the converse relationship was not apparent, with RAD21 and CTCF levels remaining unchanged in topoIIβ^−/−^ B cells.

Whilst the activity and binding of topo II was found to be dependent on cohesin, the processing of these sites was sensitive to transcriptional activity. Etoposide-induced topoIIcc occurring within transcription start sites (TSS) or gene bodies were more likely to produce chromosomal translocations than those occurring in intergenic regions, with higher levels of translocations in regions of high transcription and decreased levels of translocations on addition of the transcriptional inhibitor 5,6-dichloro-1-beta-D-ribofuranosylbenzimidazole (DRB). In eukaryotic cells, the conversion of a topoIIcc to a protein-free DSB is mediated by TDP2. The processing of topoIIcc in the presence of DRB was decreased, with more lesions remaining protein bound, whilst transcriptional activation by INFβ increased levels of protein-free DSBs. Hence, the processing and repair of topoIIcc is stimulated by transcriptional activity, and this in turn leads to an increase in translocation rate. This effect seemed to be dependent on proteasome activity, rather than TDP2, with proteasome inhibition by epoxomicin increasing the number of topoIIcc that are reversible (protein-bound) and decreasing the amount of protein-free DSBs 5.5-fold.

### 1.5. DNA Topoisomerase IV

In 2018, NGS was used to explore DNA topoisomerase IV (topo IV) binding and cleavage sites genome wide in *Escherichia coli* [16,17]. To explore binding in the absence of cleavage, a traditional ChIP-seq method was employed in which topo IV was allowed to bind DNA and then crosslinked, followed by antibody-based fragment capture. To explore topo IV cleavage complexes, a novel approach was developed, called NorflIP, relying on norfloxacin, a quinolone that traps cleavage complexes produced by prokaryotic type II topoisomerases.

Four highly enriched topo IV binding sites were found, one of which corresponded to the *dif* site, a previously confirmed hotspot of topo IV cleavage. The *dif* (deletion induced filamentation) site is located at the replication terminus and is important for chromosome dimer resolution [39]. To identify additional binding sites, the raw sequencing data were filtered for sites showing the highest Pearson correlation with the sequencing pattern at the *dif* site. This approach led to the identification of 19 additional topo IV binding sites throughout the chromosome. These sites were found frequently within intergenic regions and spanned 200 bp with no significant consensus sequence. Topo IV binding enrichment was also detected in GC rich regions. By combining this binding data with specific topo IV cleavage sites, enhanced with norfloxacin, it was found that only certain binding sites correspond to topo IV cleavage sites, indicating that not all DNA binding results in topo IV cleavage of the DNA. The *dif* site remained strong for both binding and cleavage by topo IV, as well as a site at 2.56 Mb. However, the other strong binding sites were not present in the norfloxacin-dependent cleavage data. A characteristic topo IV-dependent peak profile was found in which two 170 bp peaks were present, separated by a 130 bp reduction in sequencing coverage. This was hypothesized to be due to the presence of topo IV residues remaining covalently bound to the 5′ ends of the cut site and preventing adapter ligation and sequencing. This characteristic signal was used to algorithmically detect genome-wide topo IV cleavage sites. Using this approach 88 sites, common across three different experiments, were identified. As expected, topo IV cleavage at the *dif* site was the most enriched. Interestingly, most NorflIP sites were not observed in the ChIP-seq binding experiments, indicating that not only does binding not strongly correlate with cleavage, but that cleavage does not strongly correlate with binding. No strong consensus sequence preference for topo IV cleavage was detected, beyond a slight bias for GC dinucleotides and an increased spacing between GATC motifs around the cleavage sites.

### 1.6. DNA Gyrase

In 2018, an NGS approach was developed that allowed the precise identification of gyrase cleavage sites genome-wide in *E. coli*, facilitating in depth analysis of drug driven cleavage in vivo [18]. The method, named Topo-seq (Figure 2C), involved trapping gyrase cleavage complexes using either oxolinic acid (oxo), ciprofloxacin (cfx), or microcin B-17 (MccB17) and immunoprecipitating the resulting fragments. They then underwent a single-strand library preparation protocol that only ligates adapters to the 3′ free strand from the cut site. This method was developed to avoid issues caused by the 5′ covalent linkage to the active site tyrosine of gyrase. Proteinase K treatment leaves the phosphotyrosyl residue at the 5′ cut site, which drastically reduces ligation efficiency of the sequencing adapters. In previous studies, the use of CIP or TDP2 offered an enzymatic solution. However, the efficiency of these enzymes is not always optimal, leading to the introduction of potential artifacts. In topo-seq, this effect is mitigated entirely by solely focusing on the unbound 3′ single strand. By sequencing only these strands from either side of the cut site, the expected characteristic signal of gyrase cleavage involves a bimodal peak with a sharp 4 bp sequence loss in the center, indicative of gyrase cleavage. This unique signal allowed gyrase cleavage sites to be accurately called and subsequently analyzed.

Using this approach, Sutormin and colleagues identified 4635 gyrase cleavage sites (GCSs) when cells were treated with cfx, 5478 when treated with oxo and 732 when treated with MccB17. An in-depth comparison among these inhibitors and their cleavage sites revealed not only inhibitor-specific cleavage patterns, but also inhibitor concentration-dependent effects. Having identified a wealth of cleavage sites, robust analysis of the gyrase sequence preference was facilitated, finding the gyrase binding motif to be both extensive and degenerate. This motif is around 130 bp in length, symmetrical on either side of the cleavage site and present in all cleavage sites, regardless of the inhibitor used. It exhibits a periodic GC content fluctuation, reminiscent of the binding pattern seen for eukaryotic nucleosomes. The only inhibitor-specific differences identified were associated with the precise cleavage site. The tendency for cfx and oxo to intercalate and drive cleavage at guanine nucleotides was confirmed. However, the sequence preference for MccB17 exhibited a variable pattern and was unlike oxo and cfx. This likely reflects the fact that oxo and cfx have a different mode of action to MccB17.

Gyrase showed a strong cleavage preference for highly transcribed loci, producing the most distinct enrichment of GCSs around the rRNA operons, known to have high rates of transcription. Furthermore, the use of rifampicin, a transcriptional inhibitor, not only decreased the number of cfx-dependent cleavage sites by half, but also produced significant relocation of GCSs away from rRNA operons and decreased gyrase avoidance of poorly transcribed regions. Together, these data implicate *E. coli* gyrase directly in topology manipulation during transcription.

### 1.7. DNA Topoisomerase IA

The first type IA topoisomerase to be explored using NGS was done in concert with gyrase and RNA polymerase (RNAP) from *Mycobacterium tuberculosis* [40]. Whilst *M. tuberculosis* is a well-known and devastating human pathogen, with resistant strains beginning to dominate in some areas of the world [41], it is also a curious bacterial species in regard to topoisomerase encoding. Whilst the majority of bacterial species encode 4 topoisomerases: two type IA topoisomerases (topo I and topo III) and two type IIA topoisomerases (DNA gyrase and topo IV), *M. tuberculosis* has only one type IA (topo I) and one type IIA (DNA gyrase) [42]. The rationale behind this study was to obtain direct evidence of topo I and DNA gyrase interacting at sites of transcription, to further support the twin-supercoiled domain model of transcription and the role that topoisomerases play in removing accumulated torsional stress in the DNA caused by the transcriptional machinery. Hypothesized by Liu and Wang in 1987, the twin-supercoiled domain model describes how the separation of the DNA strands during transcription and replication would lead to positive supercoiling ahead of the protein machinery and negative supercoiling behind [43]. Indeed, numerous in vitro studies support this hypothesis, demonstrating that transcription alters DNA topology [44]; inhibition of DNA gyrase leads to positive supercoil accumulation [45] and mutation of topo I causes negative supercoil accumulation [46]. However, direct evidence of the association of bacterial topoisomerases and the transcriptional machinery was not obtained until a ChIP-seq-based protocol was implemented genome wide using *M. tuberculosis* [40]. ChIP-seq involves the crosslinking of protein to DNA, the DNA of which is enriched for fragments bound to a protein of interest, usually using a protein-specific antibody. So, the data set produced by the subsequent sequencing of this DNA library generates a binding profile for the protein, rather than a direct read out of cleavage activity.

In this study, the binding of *M. tuberculosis* gyrase and topo I were compared to the binding of RNAP. In agreement with the twin-supercoiled domain model, both topo I and gyrase were found to be specifically enriched in regions of transcriptional activity, with a high correlation with RNAP binding. As expected, RNAP was enriched at promoter sequences with topo I enrichment proximally located and gyrase enrichment both overlapping with RNAP and downstream. In addition, both gyrase and topo I were highly enriched at the origin of replication, directly implicating them in replication, whilst only gyrase displayed a binding peak at the termination site. This is likely due to *M. tuberculosis* gyrase having a dual activity of both negative supercoiling and efficient decatenation [47], the latter being a role usually fulfilled by topo IV in other bacteria.

In 2019, another study from the same group, explored topo IA activity in more depth, contrasting binding and cleavage in *Mycobacterium smegmatis*, a close relative of *M. tuberculosis* [48]. In this study, four libraries were produced, three to probe topo IA cleavage, and another for topo IA binding. The first of the topo IA cleavage protocols involved the use of a poisonous topo IA mutant, with a substitution in the topoisomerase-primase (TOPRIM) domain (D108A), previously shown to cause accumulation of cleavage complexes [49]. Once transformed into competent *M. smegmatis* cells, an increase in cleavage complexes and stimulation of the SOS response were detected, demonstrating that the mutant protein was being expressed and interacting with the genome. The second technique designed to probe topo IA cleavage implemented the use of a newly discovered topo IA inhibitor, imipramine [50]. Imipramine is a tricyclic antidepressant that has been shown to act specifically on topo IA from mycobacteria and prevent re-ligation, thereby increasing DNA cleavage complexes. The third technique, designed to probe binding, followed a traditional ChIP-seq type method. They also managed to capture wild-type topo IA cleavage sites, in the absence of inhibitors. In all four cases, topo IA-specific DNA fragments were immunoprecipitated and sequenced using the Illumina Genome Analyzer IIx.

Being able to contrast binding and cleavage, as with topo IV [16,17], revealed a similar disconnect between binding and catalytic activity. Whilst binding was shown genome wide and somewhat evenly distributed, for topo IA, the cleavage profiles were far more region specific. Multiple regions displayed topo IA binding enrichment where cleavage was completely absent. In vitro assays demonstrated that the enriched sequence was cleavable. However, topo IA inhibition by Nucleoid Associated Proteins (NAPs) measured in vitro suggests that the inhibition of topo IA cleavage in vivo could arise from the presence of NAPs. A unique feature amongst type IA topoisomerases is the mycobacterial topo IA sequence preference for both binding and cleavage, which was identified in previous in vitro studies [51,52,53]. Analysis of the genome-wide cleavage sites of *M. smegmatis* topo IA revealed a similar consensus sequence as previously established for topo IA in vitro, indicating that topo IA binds and cleaves DNA in a sequence-dependent manner in vivo.

As with the previous study looking at topo IA and gyrase co-localization in *M. tuberculosis*, topo IA in *M. smegmatis* exhibited increased occupancy and cleavage associated with RNAP and highly transcribed genes. However, in *M. smegmatis* both cleavage and binding were detected at the termination site, a result not previously seen, implicating topo IA in chromosome segregation and suggesting that, like mycobacterial gyrase, topo IA also has dual activities in both relaxation and decatenation. In most bacterial species the dedicated type IA topo involved in decatenation is topo III, an enzyme absent in mycobacterial species. In vitro assays performed in this study did indeed show that *M. smegmatis* topo IA had significant catenation and decatenation activity, and therefore may also be involved in chromosome segregation along with gyrase, in vivo.

### 1.8. DNA Topoisomerase IB

Currently only one study has mapped genome-wide activity of human topo IB, a type IB topoisomerase [19]. Using HCT116 human colon cancer cells, topo IB binding was assayed using a ChIP-seq method, and cleavage by a method termed Top1-seq. Top1-seq involved the use of camptothecin, a topo IB inhibitor, to generate covalent cleavage complexes, which were enriched using immunoprecipitation and then sequenced.

Topo IB binding enrichment was highly correlated with transcription, with 67% of DNA-bound topo IB interacting with transcribed genes both upstream of the TSS and downstream of the transcription termination site. By quantifying the transcription level of genes using RNA-seq, a method developed to allow sequencing of the transcriptome [54], increased topo IB occupancy was shown to correlate with increased levels of transcription, providing further evidence for its role in transcriptional topology regulation in eukaryotic cells, much like topo IA in prokaryotes. Comparison of the binding landscape of topo IB to RNAPII revealed their localization was highly correlated, in a manner that suggested a direct protein-protein interaction, rather than topo IB being solely bound to regions of torsional stress within the genome. To explore this, cellular extracts were subjected to a pull-down assay, which confirmed the direct interaction between topo IB and RNAPII in the absence of DNA, indicating that topo IB is in integral member of the transcriptional machinery and is recruited to sites of RNAPII engagement.

As mentioned, this study used a technique called Top1-seq, which was designed to capture catalytically engaged topo IB, using a brief camptothecin treatment. Whilst binding of topo IB was high at promoters, particularly in concert with RNAPII, catalytically engaged topo IB was diminished at TSS but enriched within gene bodies, again with a positive correlation with transcription level. Cleavage was also biased towards the template strand, indicating that topo IB activity is coupled to the movement of the transcriptional machinery. Using complementary in vitro assays, RNAPII was found to enhance topo IB relaxation activity and increase processivity under physiological ionic conditions, whilst none of the other general transcription factors seemed to have an effect on topo IB relaxation activity. The interaction between RNAPII and topo IB was mapped to the CTD of RNAPII and the NTD of topo IB. An interaction that increased the time topo IB spends in the cleaved state, enhancing the processive removal of supercoils.

## 2. Conclusions

Since the advent of NGS technologies in 2005, numerous techniques have been developed to explore genome-wide DNA binding and cleavage landscapes, which has both answered and posed new questions concerning in vivo topoisomerase behavior. Encouraging evidence in support of the widely accepted twin-domain model, has established a greater understanding of topoisomerase activity during transcription and to a lesser extent replication. Topo II and topo I in eukaryotes exhibit higher levels of activity in the vicinity of highly transcribed genes [14,19], as has also been shown for mycobacterial topo I and gyrase [48]. Topo IV, on the other hand, demonstrates a clear preference for the *dif* site in *E. coli* [16,17], supporting the idea that decatenation is its primary cellular function [55].

Using NGS as a means to explore topo cleavage across the genome, encouraged innovation in protocol development to circumvent issues which arise as a result of the covalent linkage between the protein and the DNA backbone. Proteinase K will remove the majority of the protein but will not cleave the phosphotyrosyl bond, leaving the adduct to interfere with sequencing adapter ligation. Two enzymatic solutions have been used to remove the adduct, namely CIP and TDP2, the first of which is very inefficient and the second far less commercially accessible. Using a single-strand DNA library preparation kit, as Sutormin and colleagues did when exploring DNA gyrase activity [18], offers another elegant solution by only sequencing the free 3′ DNA strand.

One particularly intriguing result has come from contrasting topoisomerase binding with cleavage activity. For some proteins, e.g., topo IV and topo IA, the correlation between them is so low that most binding sites do not constitute cleavage sites and vice versa. The nature of the techniques used to explore binding verses cleavage (such as using cleavage complex stabilizing agents which bias cleavage preference) and the platforms used to sequence the fragments, may contribute to this discrepancy. However, in vitro biochemical data also suggest that the relationship between cleavage and binding is not so clear. For example, topo II*α* whilst being more catalytically active on positively supercoiled DNA, maintains higher levels of cleavage on negatively supercoiled DNA [56]. This raises questions as to the determinants of topo binding and cleavage sites; how do the factors known to influence binding and activity including DNA topology and sequence play off against protein-mediated recruitment or inhibition in trans, and what other factors may influence topo binding and cleavage in vivo. It is clear the answer is not straightforward, with topo IB being directly recruited as part of the transcriptional machinery [19] and mycobacterial topo IA [48] and prokaryotic DNA gyrase [18] exhibiting more substantial DNA sequence preferences. As with all cellular processes, the mechanisms governing DNA topology maintenance are often not only protein specific but also species specific. A difficulty with collecting such vast amounts of data is the interpretation thereof, which has been challenging in some cases due to the complexity of the cellular environment. One possible approach would be the use of in vitro NGS techniques to map topo binding and cleavage in which DNA topology and sequence can be systematically controlled in addition to the use of specific proteins and precise buffer conditions, including inhibitor addition. Such an approach would complement genome-wide studies and could provide insights that could aid in the interpretation of genome-wide results. NGS is undoubtedly a very powerful technique, both in isolation and as part of an experimental repertoire, which will hopefully be further implemented in the field of DNA topoisomerases.

## Figures and Tables

**Table 1:** A summary of the techniques discussed in this review, which were developed to find ways to explore topo binding and cleavage activity genome wide.

Method	Protein	NGS Platform	Brief Description
Type I/II topo ChIP-seq	Type I/II topos	Non-specific	To determine the location and sequence context of topoisomerase binding to DNA using ChIP-seq. Crosslinking agent is applied to cells, before lysis, sonication, and immunoprecipitation of DNA bound to protein of interest. Crosslinking is reversed and DNA is ligated to adapters and sequenced [8].
BLESS	Non-specific	Roche 454 or Illumina Hiseq	To map the location of double strand breaks over the genome using direct in situ Breaks Labelling, Enrichment on Streptavidin and next-generation Sequencing (BLESS). Using human and mouse cells, a fixing agent is used before the cells are lysed and purified nuclei extracted. Biotinylated adapters are used to label DSBs in situ before the DNA is extracted, sonicated and enriched using streptavidin. After a second adapter ligation phase, the DNA is sequenced [9].
END-seq	Non-specific	Illumina Hiseq2500 or Illumina Nextseq500	A more sensitive and robust approach to map DSBs. Mouse lymphocytes or thymocytes are fixed in agarose before being treated with a proteinase K solution, followed by an RNase solution. The biotinylated adapters are then ligated before the DNA is extracted, sonicated and enriched using streptavidin. The DNA then undergoes a second adapter ligation before being sequenced [10]. This protocol was also adapted for use in human and mouse cells looking at topo II cleavage complexes [11].
Spo11-oligo-seq	Spo11	Roche 454	To map the location and sequence context of Spo11 mediated DNA cleavage. Nuclei from meiotic *Saccharomyces cerevisiae* cultures is extracted, sonicated and ssDNA oligos bound to Spo11 are immunoprecipitated. Proteinase K removes Spo11and DNA is extended at the 3′ end by TdT before a dsDNA adapter is ligated, and the complementary strand synthesized. Adapter 1 contains an inverted dT so the 3′ end is unmodifiable, as is the 5′ end due to the phosphotyrosyl adduct. The DNA then undergoes another 3′ extension and adapter ligation which only affects the newly synthesized strand. The dsDNA fragments are then sequenced [12].
SSB-seq and DSB-seq	Topo II	Illumina GA	To map the location and sequence context of single- and double-stranded DNA breaks induced by Topo II. Human colon cancer cells (HCT116) are treated with etoposide and the genomic DNA is extracted. For single-stranded break (SSB)-seq, SSBs are labelled using nick translation [13] involving digoxigenin labelled dUTP so that fragments can be immunoprecipitated using anti-digoxigenin. For single-stranded break (DSB)-seq, the DNA is 3′-end tailed using TdT in the presence of biotinylated dNTPs and is enriched using streptavidin coated beads. The S1-nuclease is used to cleave the 3′-end tailing and then both the SSB- and DSB-seq DNA populations are adapter ligated and sequenced [14].
CC-seq	Topo II	Illumina Miseq or Nextseq	To map the location and sequence context of Topo II cleavage complexes using cleavage complex (CC)-seq. Human or *S. cerevisiae* cells are treated with etoposide before lysis. Bulk cellular proteins are removed using a phenol-chloroform extraction and the DNA retained in the aqueous fraction is sonicated. Protein bound DNA is enriched using a silica membrane. The first adapter is ligated to the sonicated DNA-end before Topo II is removed using TDP2, followed by the second adapter ligation and sequencing [15].
NorflIP	Topo IV	Illumina GA	To map the location and sequence context of Topo IV cleavage using nofloxacin immunoprecipitation (NorflIP). *Escherichia coli* (*E. coli*) carrying C-terminal FLAG fusions of ParE and ParC is treated with norfloxacin before lysis, sonication and immune-precipitation using anti-FLAG. Adapters are ligated and fragments sequenced [16,17].
Topo-seq	DNA gyrase	Illumina Nextseq	To map the location and sequence context of DNA gyrase cleavage. *E. coli* cells are treated with ciprofloxacin, oxolinic acid or microcin B17, before lysis and sonication. DNA bound to gyrase is enriched using immunoprecipitation and proteinase K is used to remove gyrase. The DNA is then treated using the Accel 1S NGS kit, which ligates adapters to the 3′ strand from the gyrase cleavage site (5′ strand is unmodifiable due to the phosphotyrosyl adduct), and synthesizes the complementary strand. The resultant dsDNA is then sequenced [18].
Top1-seq	Topo 1B	Illumina GAII and SoLid (applied biosystems)	To map the location and sequence context of Topo 1B cleavage. Human colon cancer cells (HCT116) are briefly treated with camptothecin before being lysed, sonicated and immunoprecipitated. Topo 1B is proteolyzed and the DNA ligated to adapters and sequenced [19].
